# Towards a solution to MERS: protective human monoclonal antibodies targeting different domains and functions of the MERS-coronavirus spike glycoprotein

**DOI:** 10.1080/22221751.2019.1597644

**Published:** 2019-04-02

**Authors:** Ivy Widjaja, Chunyan Wang, Rien van Haperen, Javier Gutiérrez-Álvarez, Brenda van Dieren, Nisreen M.A. Okba, V. Stalin Raj, Wentao Li, Raul Fernandez-Delgado, Frank Grosveld, Frank J. M. van Kuppeveld, Bart L. Haagmans, Luis Enjuanes, Dubravka Drabek, Berend-Jan Bosch

**Affiliations:** a Virology Division, Department of Infectious Diseases & Immunology, Faculty of Veterinary Medicine, Utrecht University, Utrecht, Netherlands; b Department of Cell Biology, Erasmus MC, Rotterdam, Netherlands; c Harbour Antibodies B.V., Rotterdam, Netherlands; d Department of Molecular and Cell Biology, National Center for Biotechnology-Spanish National Research Council (CNB-CSIC), Madrid, Spain; e Department of Viroscience, Erasmus Medical Center, Rotterdam, Netherlands

**Keywords:** Coronavirus, MERS, antibodies, spike protein

## Abstract

The Middle-East respiratory syndrome coronavirus (MERS-CoV) is a zoonotic virus that causes severe and often fatal respiratory disease in humans. Efforts to develop antibody-based therapies have focused on neutralizing antibodies that target the receptor binding domain of the viral spike protein thereby blocking receptor binding. Here, we developed a set of human monoclonal antibodies that target functionally distinct domains of the MERS-CoV spike protein. These antibodies belong to six distinct epitope groups and interfere with the three critical entry functions of the MERS-CoV spike protein: sialic acid binding, receptor binding and membrane fusion. Passive immunization with potently as well as with poorly neutralizing antibodies protected mice from lethal MERS-CoV challenge. Collectively, these antibodies offer new ways to gain humoral protection in humans against the emerging MERS-CoV by targeting different spike protein epitopes and functions.

## Introduction

Middle-East respiratory syndrome coronavirus (MERS-CoV) is an emerging zoonotic virus that causes severe and often fatal respiratory illness in humans. Since its first identification in Saudi Arabia in 2012, the MERS-CoV documented infections in humans steadily increased, with 2298 cases as of February 2019 with an estimated 35% lethality [1]. Dromedary camels are the natural reservoir of MERS-CoV from which zoonotic transmission can occur. A vast majority of dromedary camels in the Arabian Peninsula appeared to be seropositive for MERS-CoV and MERS-CoV strains found in epidemiologically-linked humans and dromedary camels are nearly identical [2–5]. Human-to-human transmission is inefficient but can occur upon close contact such as in household or hospital settings among patients and from patients to health-care workers [2]. The high rate of MERS-CoV infection in the dromedary camel reservoir poses a persistent threat for reintroduction of MERS-CoV into humans.

Despite its continuous threat to public health, antiviral therapies or vaccines to treat or prevent MERS-CoV infection are currently lacking. The viral spikes on the surface of the enveloped MERS-CoV virions are the primary antigenic target for the development of vaccines and antibody therapies [6]. These spikes mediate virus entry into host cells and consist of three spike (S) glycoproteins, each containing the receptor binding subunit S1 and the membrane-anchored fusion-mediating subunit S2. Single-particle cryo-electron microscopic analyses of trimeric S ectodomains of several coronaviruses, including MERS-CoV [7–13], revealed a multi-domain architecture of the S1 subunit, consisting of four core domains (designated A though D). For MERS-CoV, two of these spike domains engage with host molecules to ensure entry into target cells. The S1^B^ domain of the MERS-CoV spike protein binds the host receptor DPP4 which is essential for cell entry [14,15], whereas the N-terminal domain S1^A^ binds to sialoglycans on mucins and on the host cell surface, which enhances infection of MERS-CoV on human lung cells [16]. Three S2 subunits form the stem of the spikes, which can undergo extensive conformational changes enabling membrane fusion. The surface of the S2 subunit – to which antibodies bind – shows a higher degree of sequence conservation than the more variable S1 subunit [7].

Several potently neutralizing human monoclonal antibodies against MERS-CoV have been developed using various approaches including single cell culturing methods of memory B cells isolated from MERS-CoV patients [17], hybridoma fusion of B-cells from immunized transgenic mice encoding human variable immunoglobulin domains [18–20], and phage or yeast display screening of Fab fragments of non-immune human antibody libraries [21–23]. Some of these antibodies showed protection against MERS-CoV challenge in animal models [17, 19–22, 24–27]. All of these antibodies were selected based on their *in vitro* neutralizing capacity and most of them targeted the MERS-CoV S1^B^ receptor binding domain (RBD). Structural and functional studies indicate that the epitopes of those S1^B^-specific antibodies overlap with the DPP4 binding site, explaining their potent neutralizing capacity [17, 19, 20, 28, 29]. So far only two murine monoclonal antibodies have been described that target the MERS-CoV S outside the RBD [29]. Using a combination of DNA and protein vaccination of mice, Wang et al, described S1-specific non-RBD and S2-specific neutralizing murine antibodies that showed potency for protection against MERS-CoV *in vivo* [30]. Although RBD-specific antibodies are undoubtly very potent in neutralizing MERS-CoV, effective antibody therapies are likely to require the combination of neutralizing and non-neutralizing antibodies, targeting multiple epitopes and exhibiting diverse mechanisms of actions, including Fc-mediated antibody effector functions [31–33]. In addition, such antibodies should preferably be human to avoid an immune reaction against anti-MERS antibodies of a different species. Identification of such protective antibodies, their targets and mechanisms of activity is important for developing antibody-based therapies against MERS-CoV.

In this study, we developed a set of human monoclonal antibodies against MERS-CoV with diverse mechanisms of action that show protective efficacy *in vivo*. We used the transgenic H2L2 mice encoding the human immunoglobulin variable regions to generate antibodies targeting the MERS-CoV spike protein (MERS-S). From a large panel of MERS-S-specific H2L2 antibodies, eight fully human monoclonal antibodies were generated that bind non-overlapping epitopes on MERS-S with high affinity and interfere with the three known functions of the viral protein: sialic acid binding, receptor binding and membrane fusion. These antibodies were shown to protect mice from a lethal MERS-CoV infection at low dosage. These studies extend our knowledge on the protective value of monoclonal antibodies targeting – non-RBD – domains of MERS-S.

## Results

### Isolation of H2L2 antibodies targeting different domains of the MERS-CoV spike protein

**Figure 1.**
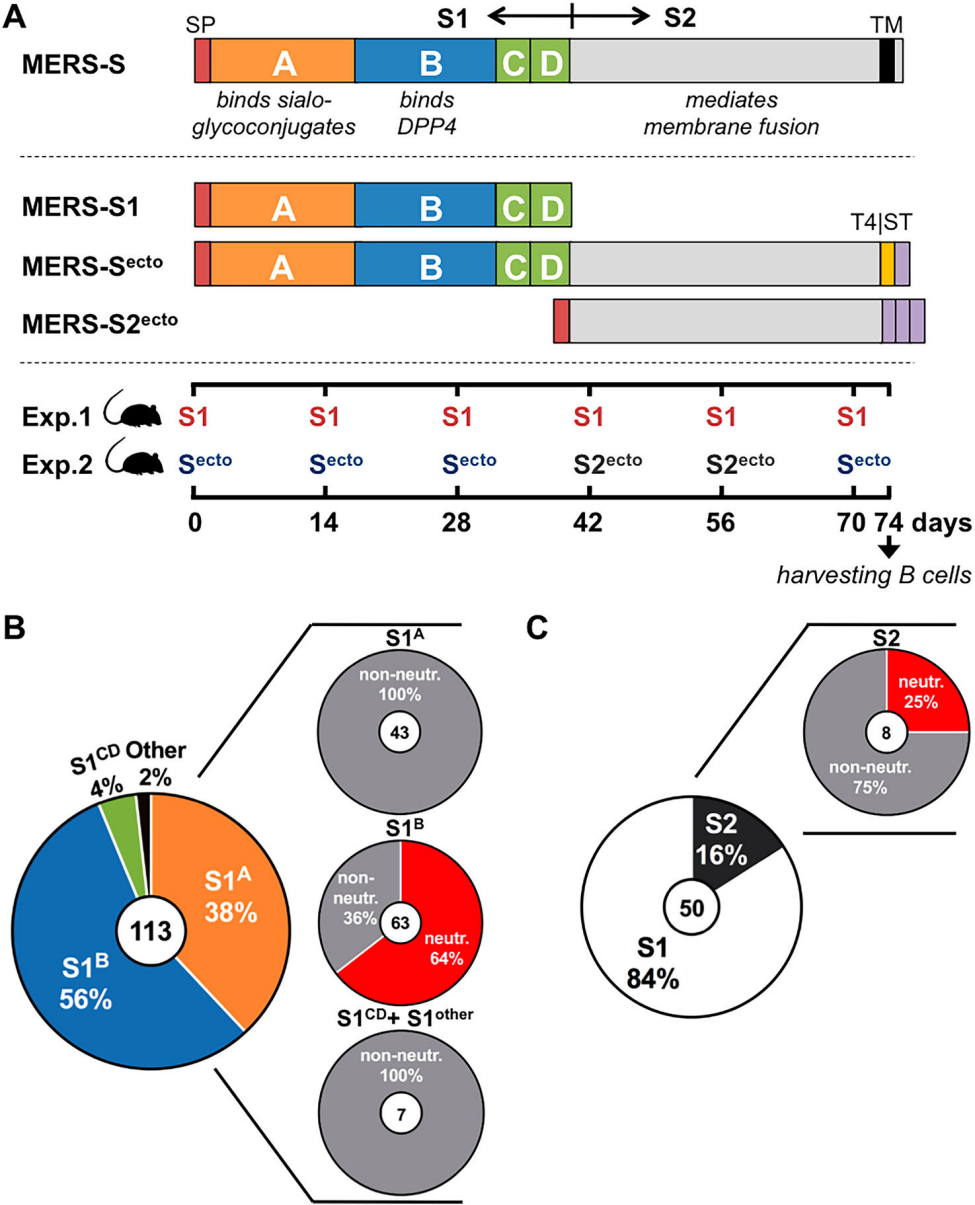
Generation and characterization of monoclonal antibodies targeting the MERS-CoV spike protein. (A) The MERS-CoV spike (S) protein and recombinant soluble MERS-CoV S antigens used for immunization of H2L2 transgenic mice to generate human monoclonal antibodies (mAbs). Upper panel: Schematic representation of the MERS-CoV S protein, indicated are S subunits (S1 and S2), S1 domains (A through D), and known biological functions. Middle panel: Schematic representation of recombinant soluble MERS-CoV S antigens, including the MERS-CoV S S1 subunit (MERS-S1), the ectodomain of its S2 subunit (MERS-S2^ecto^) or the entire MERS-S ectodomain (MERS-S^ecto^), the latter containing a mutation at the furin cleavage site at the S1/S2 junction and a C-terminally fused T4 foldon trimerization tag to increase trimer stability (T4). Positions of signal peptides (SP) and StrepTag affinity tags (ST) are indicated. Lower panel: Immunization schedule H2L2 mice. To generate monoclonal antibodies (mAbs) targeting the MERS-CoV S protein, groups of H2L2 mice (six mice/group) were immunized with either MERS-S1 (6×), or sequentially immunized with MERS-S^ecto^ (3×), MERS-S2^ecto^ (2×) and MERS-S^ecto^ (1x). Booster immunizations were done with two-week intervals and B-cells were harvested from spleen and lymph nodes four days after the last immunization. (B) Identified MERS-S1-reactive mAbs of hybridomas derived from B-cells of S1-immunized H2L2 mice were characterized for epitope location and virus neutralization using MERS-S pseudotyped VSV. Pie charts show mAb frequencies relative to the total (indicated in the centre circle). Domain-level epitope mapping was performed for MERS-S1-reactive mAbs and relative frequencies of mAbs binding to given S1 domains (S1^A^, S1^B^ or S1^CD^) are indicated. The percentage of mAbs that was reactive to S1 but not to the S1^A^, S1^B^ or S1^CD^ domains (S1^other^) is also shown. Virus neutralization by S1-reactive mAbs was analysed using the luciferase-encoding MERS-CoV S pseudotyped VSV particles. (C) Identified MERS-S^ecto^-reactive mAbs of hybridomas generated from S^ecto^/S2^ecto^ immunized H2L2 mice were characterized for epitope location and virus neutralization as in (B).

To further our understanding of antibodies that contribute to humoral immunity, we generated and characterized a panel of human antibodies targeting different functional domains of the MERS-CoV spike protein. To develop human monoclonal antibodies (mAbs), we employed H2L2 transgenic mice carrying immunoglobulin transgenes for human variable heavy and light chains and rodent constant regions (http://www.harbourbiomed.com). In one immunization experiment, H2L2 mice were immunized with purified MERS-S1 subunit (Figure 1(A), SI Appendix, Fig. S1A). A second immunization experiment was done to generate antibodies targeting the more conserved MERS-S2 subunit using the MERS-S ectodomain and MERS-S2 ectodomain as antigens following a sequential immunization strategy (Figure 1(A), SI Appendix, Figure S1A). Hybridoma cell lines were generated from spleen and lymph node derived B-cells from both immunization experiments and antibody-containing hybridoma supernatants were screened for MERS-S reactivity by ELISA. The first immunization experiment provided 113 hybridoma supernatants (out of 4553) that reacted to MERS-S1. To understand the immunogenicity landscape of the MERS-S1 subunit, we mapped epitopes of these antibodies to S1 domains for which individual S1 domains were expressed and used as antigens in ELISA (SI Appendix, Figure S1B). The majority of S1-reactive antibodies bound either to domain S1^B^ (56%) or S1^A^ (38%) (Figure 1(B), SI Appendix, Fig. S2A). Four percent of the S1-reactive antibodies bound to either domain S1^C^ or S1^D^, whereas some antibodies (2%) did not bind to any of the S1 domains, indicative of binding to interdomain epitopes (Figure 1(B), SI Appendix, Figure S2A). The second immunization with MERS-S ectodomain and MERS-S2 ectodomain resulted in 50 hybridoma supernatants (out of 1158 hybridomas) that were positive for binding in a MERS-S ectodomain ELISA. Most of the MERS-S reactive antibodies bound to S1 (84%) and eight (16%) were found to bind S2 (Figure 1(B)). Virus neutralization activity of hybridoma supernatants was screened using luciferase-encoding MERS-S pseudotyped vesicular stomatitis virus. Forty of the 113 MERS-S1-specific antibodies from MERS-S1-immunized mice neutralized MERS-CoV infection (Figure 1(B), SI Appendix, Figure S2A). Notably, all of the neutralizing antibody epitopes mapped to the receptor binding domain S1^B^. Two out of the eight identified MERS-S2-specific antibodies were found to neutralize MERS-S pseudovirus (Figure 1(B), SI Appendix, Figure S2C). Screening the neutralizing antibodies for antigen binding competition identified six epitope groups, which was used for the selection of lead antibodies (SI Appendix, Figure S2B).

### Binding of lead human mAbs to the MERS-CoV spike protein

From the set of MERS-CoV-S specific H2L2 antibodies, a panel of eight monoclonal antibodies (mAbs 1.10f3, 7.7g6, 1.6f9, 1.2g5, 1.8e5, 4.6e10, 1.6c7 and 3.5g6) were selected with epitopes distributed throughout different domains of the MERS-CoV spike protein for further detailed biophysical and functional characterization. Selection of H2L2 mAbs was based on their unique variable heavy and light chain sequences, and on their capacity to neutralize MERS-CoV relative to other mAbs within an epitope group (SI Appendix, Figure S2C). We could not detect MERS-S1^A^-specific neutralizing antibodies but we nevertheless selected one non-neutralizing mAb (1.10f3) that recognizes this sialic acid-binding domain. Fully human mAbs were generated by cloning the genes of the variable region of light and heavy chain into human IgG1 expression vectors. Likewise, IgG1 expression vectors were generated for the expression of a previously reported potent MERS-CoV-neutralizing antibody (anti-MERS control) as a benchmark antibody [34]. In addition, we used an irrelevant antibody recognizing the Strep-tag affinity tag (isotype control). All reformatted antibodies were expressed in human HEK-293 T cells and purified using Protein-A affinity purification (SI Appendix, Figure S3).

**Figure 2.**
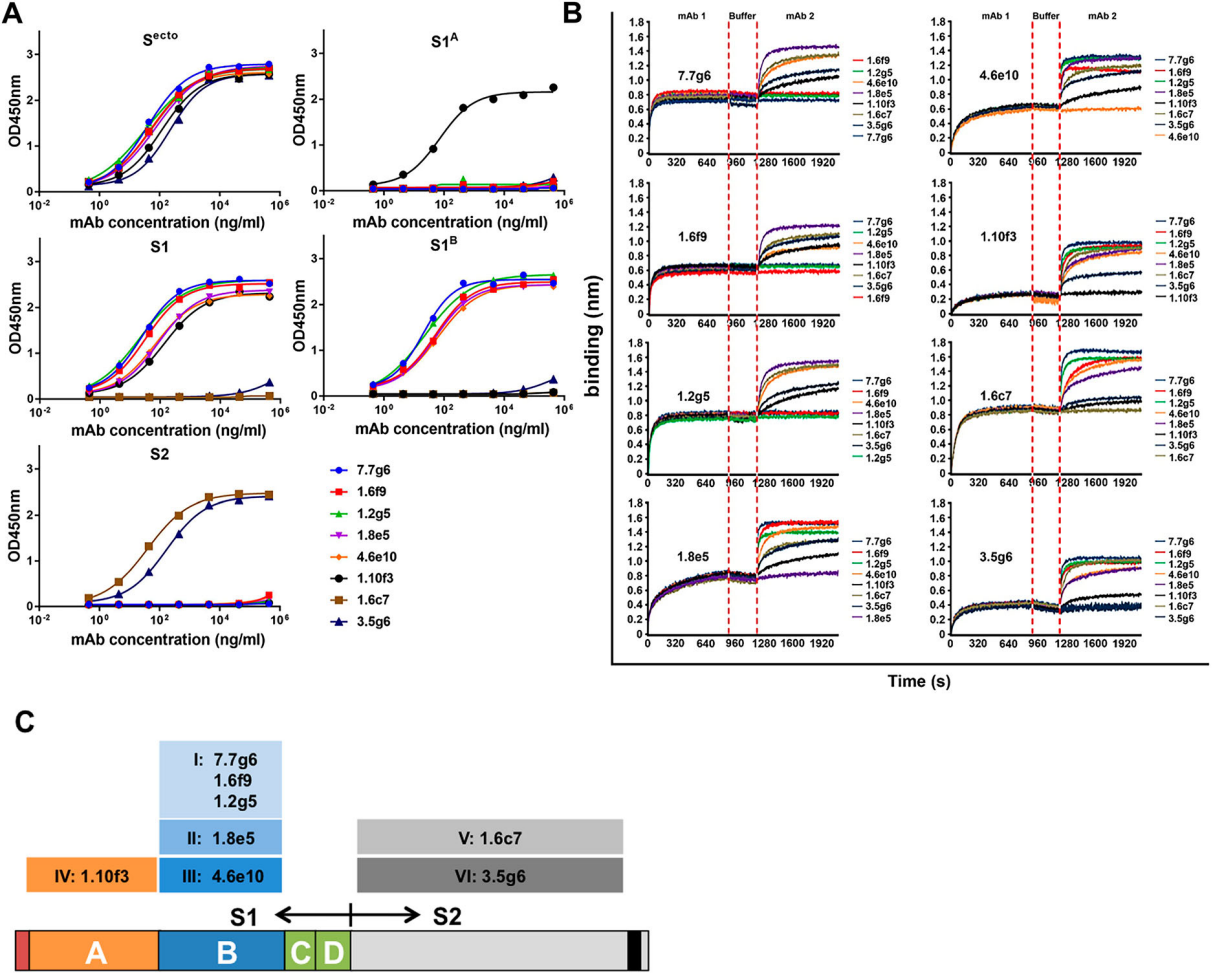
Human anti-MERS-S mAbs targeting six epitope groups distributed over multiple domains of the MERS-CoV spike protein. (A) ELISA reactivity of the human anti-MERS-S mAbs to the indicated MERS-CoV spike glycoprotein domains. (B) Binding competition of anti-MERS-S mAbs analysed by bio-layer interferometry (BLI). Immobilized MERS-S^ecto^ antigen was saturated in binding with a given anti-MERS-S mAb (step 1) and then exposed to binding by a second mAb (step 2). Additional binding of the second antibody indicates the presence of an unoccupied epitope, whereas lack of binding indicates epitope blocking by the first antibody. As a control, the first mAb was also included in the second step to check for self-competition. (C) Schematic distribution of epitope groups of anti-MERS-S mAbs over the different MERS-S domains.

Epitope mapping of purified human mAbs to the different domains of MERS-CoV S was done by ELISA using soluble MERS-CoV S^ecto^, S1, S1^A^, S1^B^ or S2^ecto^ as antigens (Figure 2(A), SI Appendix, Figure S1B). Domain-level epitope mapping confirmed that mAb 1.10f3 bound to the sialic acid binding domain S1^A^, mAbs 7.7g6, 1.6f9, 1.2g5, 1.8e5 and 4.6e10 targeted the receptor binding domain S1^B^ whereas mAbs 1.6c7 and 3.5g6 bound the ectodomain of the membrane fusion subunit S2 (Figure 2(A)). Next competition for binding of the lead antibodies to the MERS-S ectodomain was tested using bio-layer interferometry (Figure 2(B)). The binding competition data indicated the existence of six epitope groups suggesting the presence of six distinct epitopes targeted by the eight lead mAbs on the MERS-CoV S protein: group I (7.7g6, 1.6f9 and 1.2g5), group II (1.8e5) and group III (4.6e10) on the S1^B^ domain, group IV (1.10f3) on the S1^A^ domain, and groups V (1.6c7) and group VI (3.5g6) on the S2 ectodomain. Antibodies of different groups competed minimally with each other, indicating that their epitopes were largely distinct (Figure 2(B) and (C)). The binding kinetics of the eight human monoclonal antibodies was determined by bio-layer interferometry. Strep-tagged MERS-S ectodomain was captured on the protein-A sensor via an anti-Streptag antibody and kinetic binding parameters of antibodies were determined at 25°C and pH 7.4. All antibodies displayed high affinity binding for the MERS-S ectodomain with equilibrium dissociation constants (*K*_D_) in the nano- to the picomolar range (0.081 to 4.78 nM) (Table 1, SI Appendix, Figure S4). The binding affinity of the MERS-CoV receptor DPP4 to MERS-S was measured in the same set up, and was lower compared to the binding affinity of the mAbs that target the receptor binding domain S1^B^ (Table 1, SI Appendix, Figure S4).

**Table 1.** Binding kinetics of mAbs/MERS-S^ecto^ or DPP4/MERS-S^ecto^ from bio-layer interferometry measurements.

mAb	*K_D_ *(M)	*k_on_ *(M^−1^ sec^−1^)	*k_off_* (sec^−1^)
7.7g6	3.612 × 10^−10^	3.196 × 10^4^	1.154 × 10^−5^
1.6f9	5.298 × 10^−10^	1.175 × 10^4^	6.227 × 10^−6^
1.2g5	8.086 × 10^−11^	7.587 × 10^4^	6.134 × 10^−6^
1.8e5	3.178 × 10^−10^	2.442 × 10^4^	4.232 × 10^−6^
4.6e10	3.592 × 10^−10^	3.569 × 10^4^	1.134 × 10^−5^
1.10f3	4.784 × 10^−9^	9.550 × 10^3^	4.569 × 10^−5^
1.6c7	5.007 × 10^−10^	5.927 × 10^4^	2.968 × 10^−5^
3.5g6	2.246 × 10^−9^	2.107 × 10^4^	4.733 × 10^−5^
α-MERS-CTRL	1.289 × 10^−10^	4.576 × 10^4^	5.900 × 10^−6^
DPP4	3.416 × 10^−9^	1.423 × 10^4^	4.859 × 10^−5^

### Anti-MERS-S mAbs bind cell surface displayed MERS-CoV spike protein

To assess whether the lead mAbs can bind full-length MERS-CoV S expressed on the cell surface, we transfected Huh-7 cells with plasmid encoding MERS-CoV S. The spike gene was C-terminally extended with GFP to monitor MERS-S expression, and mutated at the furin cleavage site to stabilize the spike protein in its native prefusion state and to prevent MERS-S-mediated cell–cell fusion. Binding of lead mAbs to cell-surface expressed MERS-S was analysed by flow cytometry and immunofluorescence. All anti-MERS-S mAbs bound to non-permeabilized, MERS-S transfected (GFP-positive) Huh-7 cells in both assays, indicative for binding to cell surface displayed MERS-CoV S (SI Appendix, Figures S5 and S6).

### Neutralization activity of anti-MERS-S mAbs

**Figure 3.**
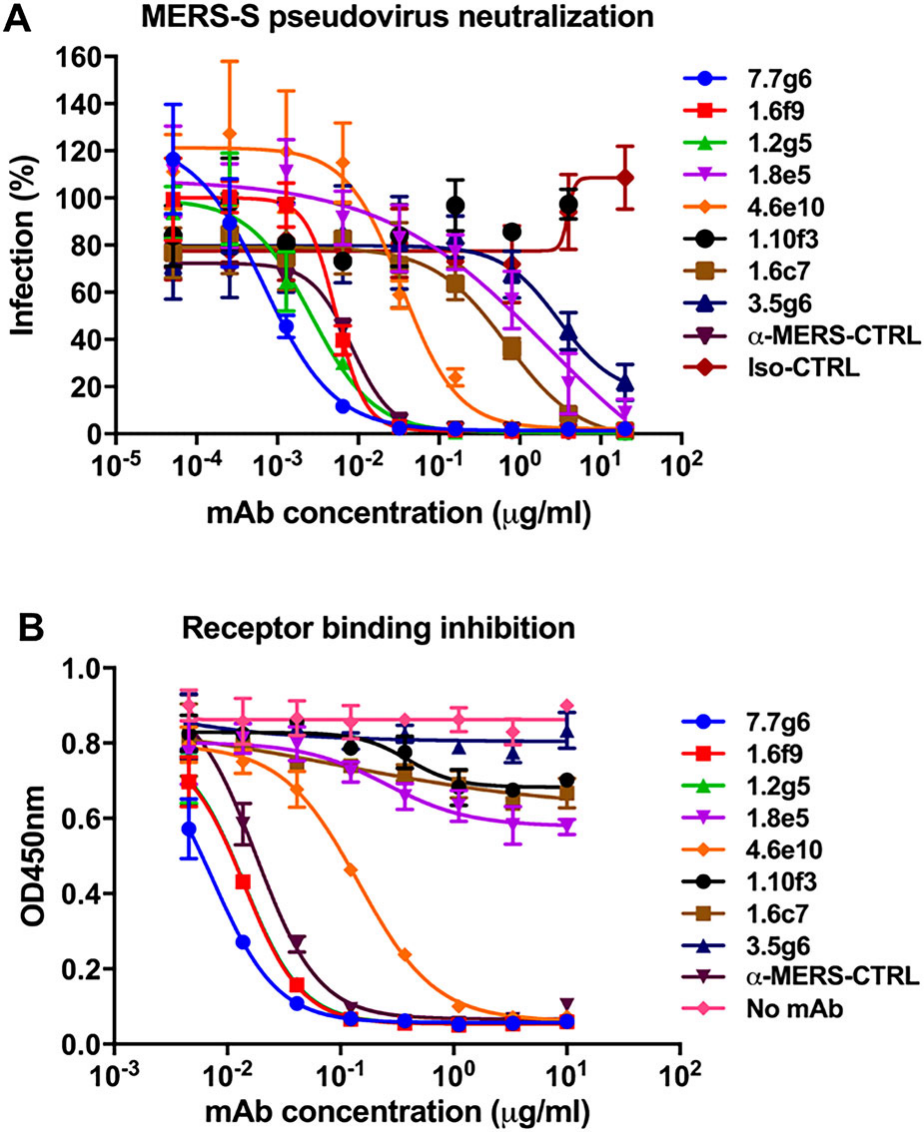
Virus neutralization and receptor binding inhibition by anti-MERS-S mAbs. (A) Analysis of MERS-CoV neutralizing activity by anti-MERS-S mAbs using MERS-S pseudotyped, luciferase-encoding VSV. A previously described RBD-specific, MERS-CoV-neutralizing human monoclonal antibody (anti-MERS-CTRL) and irrelevant isotype monoclonal antibody (Iso-CTRL) were included as positive and negative control, respectively. Luciferase-expressing VSV particles pseudotyped with the MERS-CoV S protein or authentic MERS-CoV were incubated with antibodies at the indicated concentrations and the mix was used to transduce Huh-7 cells. At 24 h postinfection luciferase expression was measured and neutralization (%) was calculated as the ratio of luciferase signal relative to relative to non-antibody-treated controls. Data represent the mean (± standard deviation, SD) of three independent experiments. (B) Receptor binding inhibition by anti-MERS-S mAbs, determined by an ELISA-based assay. Recombinant soluble MERS-S^ecto^ was preincubated with serially diluted anti-MERS-S mAbs and added to ELISA plates coated with soluble the MERS-CoV S ectodomain. Binding of MERS-S^ecto^ to DPP4 was measured using HRP-conjugated antibody recognizing the Streptag affinity tag on DPP4. Data represent the mean (± standard deviation, SD) of three independent experiments.

The ability of human lead mAbs to neutralize MERS-CoV infection *in vitro* was tested on Vero cells with luciferase-encoding MERS-S pseudotyped virus, and with authentic MERS-CoV using a plaque reduction neutralization test (PRNT). Levels of virus neutralization varied among the individual antibodies (Figure 3(A) and Table 2). The 7.7g6, 1.6f9 and 1.2g5 mAbs all targeting epitope group I on MERS-S1^B^ showed the most potent neutralizing activity, and displayed picomolar half-maximal inhibitory concentrations against MERS-S pseudotyped virus (IC_50 _= 7–30 pM) and authentic MERS-CoV (PRNT_50 _= 53–200 pM), which was equivalent to or lower than our benchmark MERS-S neutralizing monoclonal antibody that targets the same domain (Table 2). MERS-S1^B^ mAbs from epitope group II (mAb 1.8e5) and III (mAb 4.6e10) neutralized MERS-S pseudovirus infection at nanomolar concentrations (IC_50_ = 10 and 0.32 nM, resp.), and exhibited no detectable or moderate neutralizing activity against authentic MERS-CoV (PRNT_50_ = > 6.67 and 6.67 nM, resp.). The MERS-S1^A^-specific mAb 1.10f3 lacked MERS-CoV neutralization activity in both virus neutralization assays. The anti-MERS-S2 mAbs 1.6c7 and 3.5g6 were able to neutralize MERS-S pseudovirus (IC_50_ = 2.45 and 16.6 nM, resp.), albeit at higher concentrations than the most potent neutralizing MERS-S1^B^ mAbs (about 100-fold higher). The isotype control did not show any neutralization in both assays. Collectively, our data demonstrate that antibodies targeting the receptor binding domain S1^B^ of the MERS-CoV spike protein display the highest potential for neutralization of MERS-CoV infection *in vitro*.

**Table 2.** Virus neutralization and receptor binding inhibition by anti-MERS-S mAbs.

mAb	MERS-S target	IC_50_* MERS-S VSVpp		PRNT_50_** MERS-CoV		RBI_50_***	
		μg/ml	nM	μg/ml	nM	μg/ml	nM
7.7g6	S1^B^	0.001	0.007	0.008	0.053	0.007	0.047
1.6f9		0.006	0.04	0.03	0.200	0.013	0.087
1.2g5		0.002	0.013	0.03	0.200	0.014	0.093
1.8e5		1.500	10	>1	>6.67	>10	>66.7
4.6e10		0.048	0.320	1	6.667	0.137	0.913
1.10f3	S1^A^	>10	> 66.7	>1	>6.67	>10	>66.7
1.6c7	S2^A^	0.367	2.447	1	6.67	>10	>66.7
3.5g6		2.488	16.6	>1	>6.6	>10	>66.7
α-MERS-CTRL	S1^B^	0.005	0.033	0.03	0.200	0.022	0.147
Iso-CTRL	–	>10	> 66.7	>1	>6.67	–	–

* IC_50_: mAb concentration resulting in half-maximal infection of MERS-S VSV pseudovirus (MERS-S VSVpp) on Vero cells.

** PRNT_50_: highest mAb dilution resulting in > 50% reduction in the number of MERS-CoV infected Vero cells.

*** RBI_50_: mAb concentration of that gives half-maximal receptor binding.

### Anti-MERS-S1^B^ mAbs neutralize MERS-CoV by blocking receptor binding

To understand the mechanism of action of lead mAbs, we set up assays to assess antibody interference with the diverse functions of the MERS-CoV S domains. To assess whether antibodies can compete with viral binding to the host receptor DPP4, we developed an ELISA-based receptor binding inhibition assay, in which binding of MERS-S ectodomain to DPP4-coated ELISA plates is quantified and interference with receptor binding by antibodies is measured as a reduction in binding signal. In the absence of antibodies, the MERS-S ectodomain showed stable binding to DPP4 (Figure 3(B)). Whereas anti-MERS-S1^B^ mAb 1.8e5 showed weak interference with binding of MERS-S ectodomain to DPP4, all other MERS-S1^B^-specific mAbs (mAbs 7.7g6, 1.6f9, 1.2g5, 4.6e10 and anti-MERS-CTRL) potently inhibited binding of MERS-S ectodomain to DPP4 in a concentration-dependent manner (Figure 3(B)). The data indicate that these antibodies partly overlap with or bind sufficiently close to the receptor-binding site on S1^B^ to compete with receptor binding. None of the antibodies that bind outside the RBD domain (MERS-S1^A^ and -S2) could block receptor binding. The potency of the S1^B^-specific mAbs to inhibit receptor binding corresponds with the ability of these antibodies to neutralize virus infection (Table 2), indicating that the inhibition of virus–receptor interaction by these antibodies is their main mechanism of neutralization *in vitro*.

### Anti-MERS-S1^A^ mAb 1.10f3 blocks binding of MERS-S1^A^ to sialoglycoconjugates

**Figure 4.**
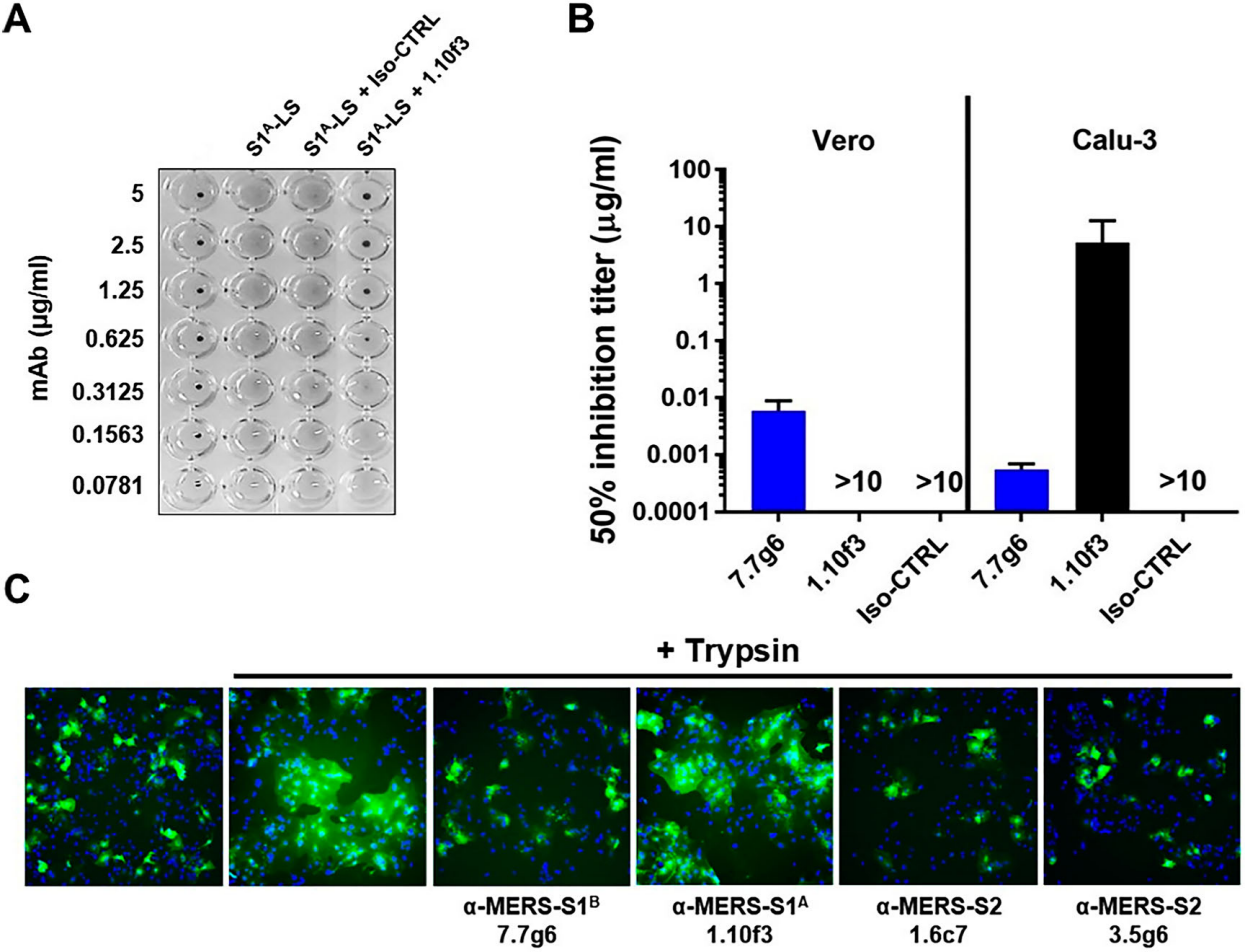
Anti-MERS-S mAbs targeting MERS-S1^A^ and -S2 domains block domain-specific functions. (A) The anti-MERS-S1^A^ mAb 1.10f3 interferes with MERS-S1^A^-mediated sialic acid binding, determined by a hemagglutination inhibition assay [16]. The sialic-acid binding domain S1^A^ of MERS-S was fused to lumazine synthase (LS) protein that can self-assemble to form 60-meric nanoparticle (S1^A^-LS), which enables multivalent, high affinity binding of the MERS-S1^A^ domain to sialic acid ligands such as on erythocytes. Human red blood cells were mixed with S1^A^-LS in the absence or presence of 2-fold dilutions of the MERS-S1^A^-specific mAb 1.10f3. Isotype control antibody was included as a negative control. Hemagglutination was scored after 2 h of incubation at 4°C. The hemagglutination inhibition assay was performed three times, a representative experiment is shown. (B) Neutralization of MERS-S pseudotyped VSV by anti-MERS-S mAb 1.10f3 on Vero and Calu-3 cells. Data represent the mean (± standard deviation, SD) of three independent experiments. (C) The anti-MERS-S2 mAbs 1.6c7 and 3.5g6 block MERS-S-mediated cell–cell fusion. Huh-7 cells were transfected with plasmid expressing MERS-CoV S, C-terminally fused to GFP. Two days after transfection, cells were treated with trypsin to activate membrane the fusion function of the MERS-CoV S protein, and incubated in the presence or absence of anti-MERS-S2 mAbs 1.6c7 and 3.5g6, or the anti-MERS-S1^B^ mAb 7.7g6 and anti-MERS-S1^A^ 1.10f3, all at 10 μg/ml. Formation of MERS-S mediated cell–cell fusion was visualized by fluorescence microscopy. Merged images of MERS-S-GFP expressing cells (green) and DAPI-stained cell nuclei (blue) are shown. Experiment was repeated two times and representative images are shown.

Recently we demonstrated that the MERS-S1^A^ domain facilitates viral binding to cell-surface sialoglycoconjugates, which can serve as a cell attachment factor for MERS-CoV [16]. We assessed whether the MERS-S1^A^-targeting mAb 1.10f3 can interfere with binding of MERS-S1^A^ to sialoglycoconjugates on the surface of erythrocytes using the hemagglutination inhibition assay. To this end, we used lumazine synthase (LS) nanoparticles multivalently displaying MERS-S1^A^ (S1^A^-LS), which were earlier employed to demonstrate the sialic-acid dependent hemagglutination by the MERS-S1^A^ domain [16]. Hemagglutination was observed when S1^A^-LS was mixed with erythrocytes (Figure 4(A)). S1^A^-LS mediated hemaglutination was abrogated upon addition of the MERS-S1^A^ mAb 1.10f3, but not upon addition of the isotype control. Next, we assessed whether interference of 1.10f3 with binding to sialylated receptors could inhibit Sia-dependent MERS-CoV infection. Binding to sialoglycans may aid MERS-CoV entry into DPP4-positive cells, depending on the cell type. Infection of Vero cells does not depend on cell surface sialic acids, concurrent with a low abundancy of the MERS-CoV S1^A^ glycotopes on those cells [16]. Correspondingly, infection of Vero cells with MERS-S pseudovirus could not be inhibited by 1.10f3 (Figure 4(B)). By contrast, infection of human lung Calu-3 cells was shown to rely on cell surface sialic acids which correlated with the abundance of MERS-CoV S1^A^ receptors [16]. Contrary to Vero cells, infection of Calu-3 cells could be inhibited by 1.10f3 (Figure 4(B)) suggesting that antibody binding to MERS-S1^A^ can neutralize MERS-CoV infection via inhibition of virus binding to cell surface sialoglycans.

### Anti-MERS-S2 mAbs interfere with MERS-S mediated membrane fusion

The coronavirus S2 subunit encompasses the machinery for fusion of viral and host cell membranes, a process that is driven by extensive refolding of the metastable prefusion S2 into a stable postfusion state [35]. We hypothesized that antibodies targeting the MERS-S2 subunit might neutralize MERS-CoV infection by inhibiting this fusion process. To test this, we developed a MERS-CoV-S driven cell–cell fusion assay using a modified MERS-CoV spike protein. To monitor expression of MERS-CoV S in cells, we extended the viral fusion protein C-terminally with GFP. In addition, we mutated the furin cleavage site at the S1/S2 junction to increase the dependency of MERS-CoV S fusion activation on exogenous addition of trypsin. Expression of this MERS-S variant upon transfection of DPP4-expressing Huh-7 cells could be readily observed by the GFP signal (Figure 4(C)). Upon addition of trypsin, large GFP-fluorescent syncytia were detected indicating MERS-S mediated cell–cell fusion (Figure 4(C)). As expected, the addition of anti-MERS-S1^B^ (7.7g6) blocked the formation of syncytia since cell–cell fusion is dependent on receptor interaction. No effect on syncytium formation was seen for the MERS-S1^A^ mAb 1.10f3. In contrast, the MERS-S2-specific mAbs 1.6c7 and 3.5g6 both blocked syncytium formation. Since both neutralizing antibodies did not interfere with receptor binding (Figure 3(B)), we surmise that binding of these S2-specific antibodies inhibits infection by preventing conformational changes in the S2 subunit of the MERS-CoV spike protein that are required for fusion.

### Protective activity of mAbs from lethal MERS-CoV challenge

**Figure 5.**
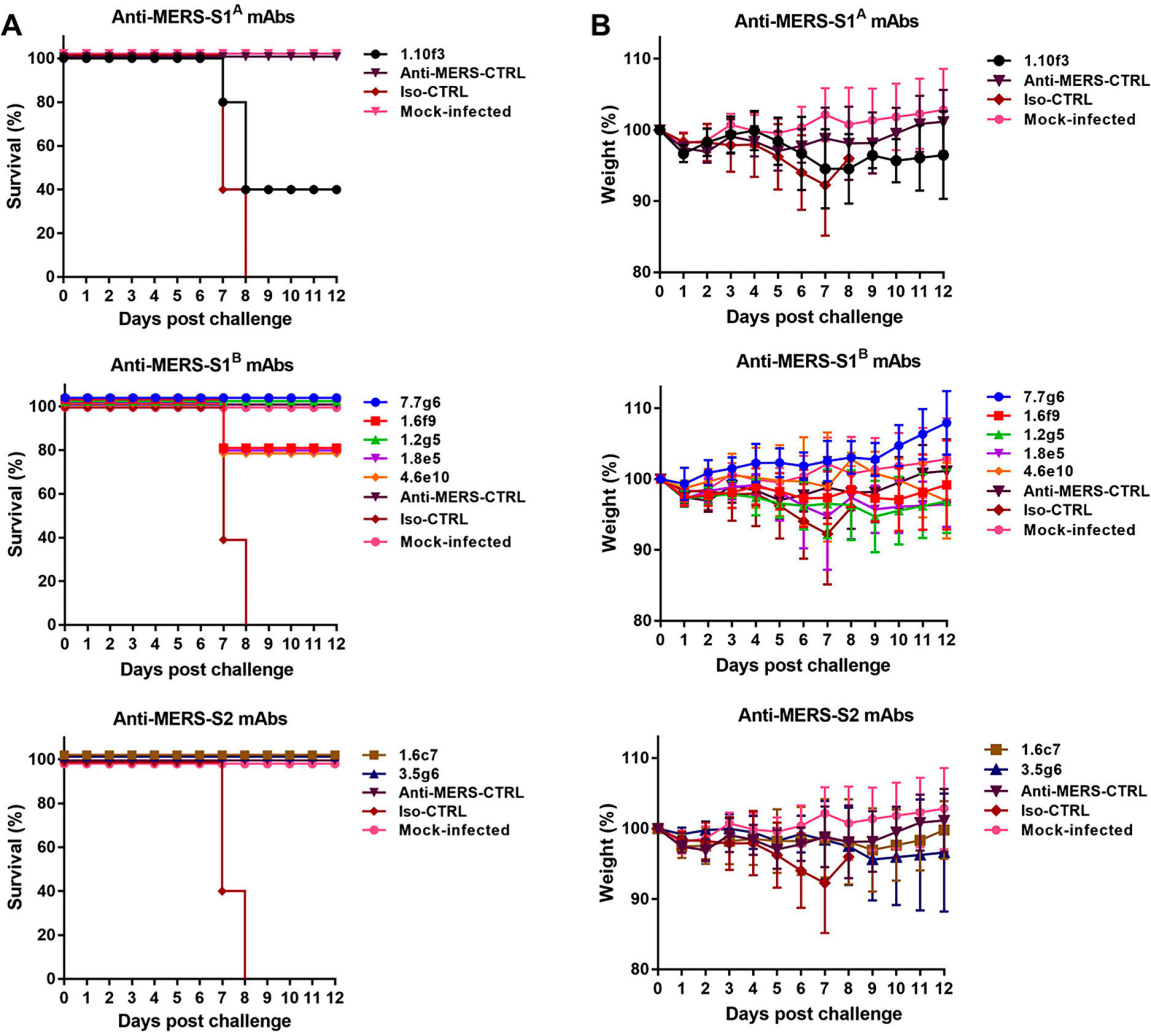
Human anti-MERS-S mAbs protect mice against lethal MERS-CoV challenge. (A–B) Fifty microgram of antibody (equivalent to 1.8 mg mAb/kg body weight) was infused intraperitoneally in K18-*hDPP4*-transgenic mice 6 h before challenge with 5 × 10^3^ pfu/mice of MERS-CoV. Five mice per group were used in the experiment. Survival rates (A) and weight loss (B) (expressed as a percentage of the initial weight) was monitored daily until 12 days post-inoculation.

To assess the prophylactic efficacy of our lead mAbs against MERS-CoV infection *in vivo*, we used transgenic K18-*hDPP4* mice expressing human DPP4 [36]. Six hours prior to MERS-CoV infection, mice (5 mice/group) were injected intraperitoneally with a 50 μg dose of each mAb (equivalent to 1.8 mg mAb per kg body weight). The percentage of survival and weight change following challenge was monitored for 12 days. MERS-CoV infection was consistently lethal as all mice that received the monoclonal isotype control showed significant weight loss and had succumbed to the infection between 7 and 8 days post-challenge (Figure 5(A) and (B)). Contrarily, all MERS-S1^B^ binding mAbs showed high levels of protection against lethal MERS-CoV challenge (80–100%, Figure 5). Anti-MERS-S1^B^ mAbs 7.7g6, 1.2g5 and the benchmark anti-MERS control mAb uniformly protected animals from death, whereas the MERS-S1^B^ mAbs 1.6f9, 1.8e5 and 4.6e10 protected 4 out of 5 animals (80%) in this model. The MERS-S1^A^ binding mAb 1.10f3 afforded partial protection from mortality (40%). Notably, the anti-MERS-S2 mAbs 1.6c7 and 3.5g6 protected all five animals from lethal infection. Relative to the isotype control treated mice, mice treated with MERS-S specific antibodies showed reduced weight loss (Figure 5(B)). These results highlight that antibodies targeting non-RBD domains (i.c. S1^A^ and S2) of the MERS-CoV spike protein can contribute to humoral immunity against MERS-CoV infection.

### Discussion

The recurring spillover infections of MERS-CoV in humans from its dromedary camel reservoir, the high mortality and person-to-person transmission pose a significant threat to public health. As there are currently no licensed vaccines or treatments for combatting MERS-CoV infections, the development of effective counter measures is a critical need, and recently prioritized by the World Health Organization in the research and development Blueprint for action to prevent epidemics [37]. Antibody therapies targeting critical entry functions of viral glycoproteins are increasingly recognized as promising antiviral strategies to protect humans from lethal disease [38]. For MERS-CoV, passive immunization studies with neutralizing antibodies in small animals support the idea that antibody therapies may hold promise to protect humans from lethal MERS-CoV mediated disease. Most of these protective antibodies neutralize virus infection by occupying the receptor binding domain (RBD) and compete with the host receptor. Here we describe the protective activity of individual human antibodies targeting RBD as well as non-RBD spike domains and their interference with the three known functions of the spike glycoprotein. Collectively, the arsenal of protective antibodies that bind to and functionally inhibit the activity of multiple spike protein domains can reveal new ways to gain humoral protection against the emerging MERS coronavirus.

Potent MERS-CoV neutralizing antibodies, as our study and that of others have shown, commonly target the S1^B^ receptor binding domain [17–23]. The anti-S1^B^ antibodies physically prevent binding to the host receptor DPP4 attributed to their higher binding affinity for MERS-S, thereby potently neutralizing MERS-CoV infection. The five S1^B^-specific antibodies identified in this study were found to target three non-overlapping epitope groups on the MERS-CoV S1^B^ domain, consistent with the three epitope groups at the spike-DPP4 receptor interface reported by Tang *et al.* [22]. These S1^B^ antibodies displayed varying neutralization potency with 100-1000-fold differences in IC_50_ values, with antibodies targeting epitope group I showing ultrapotent neutralizing activity at sub-nanomolar levels. Irrespective of the neutralizing potency, all S1^B^ antibodies displayed significant protective activity (80–100% survival rates) in mice from lethal MERS-CoV challenge.

Apart from engaging the host receptor DPP4, we earlier demonstrated that the MERS-CoV spike protein binds sialoglycoconjugates (Sia’s) on mucins and the host cell surface via an independently functional domain, the N-terminal domain S1^A^. This Sia-binding activity serves as an attachment factor for MERS-CoV infection in a cell-type dependent manner [16]. We now show that binding of the isolated mAb 1.10f3 to this MERS-S1^A^ domain abrogates Sia-binding, and can inhibit the Sia-dependent infection of human lung Calu-3 cells by MERS-CoV [16]. Moreover, passive immunization of mice with this MERS-S1^A^-specific mAb resulted in 40% protection from mortality following MERS-CoV infection. These findings underline the role of sialic acid binding for MERS-CoV infection *in vivo*, and demonstrate the importance of anti-MERS-S1^A^ antibodies in protection.

From all MERS-CoV mAbs described, only two mAbs – G2 and G4 – targeted epitopes outside the RBD [29]. G2 binds an epitope in S1 outside the RBD, whereas G4 targets a variable loop in S2 (Wang 2015). Both murine antibodies were shown to protect mice from a lethal infection of MERS-CoV *in vivo*. Both antibodies were neutralizing, though the interference of these antibodies with spike functions was not delineated. Using a tailored immunization scheme to boost antibody responses to the MERS-CoV fusion-mediating S2 subunit, we were able to recover MERS-S2-specific mAbs, two of which demonstrated neutralizing activity and fully protected mice from a lethal MERS-CoV challenge. These two S2 antibodies did not interfere with receptor binding yet abrogated cell–cell fusion, implying their interference with spike-mediated membrane fusion by preventing conformational changes required for fusion. The highly protective S2 mAbs only displayed modest *in vitro* neutralizing activity, indicating that strong neutralizing activity is not a prerequisite for protection.

Apart from interfering with functions of viral proteins, antibodies are able to employ a broad range of antiviral activities through the innate immune system. Binding of antibodies to glycoproteins on the surface of infected cells or on viruses may decorate them for destruction through Fc-mediated antiviral activities including antibody-dependent cellular cytotoxicity (ADCC), antibody-dependent cell-mediated phagocytosis (ADCP) or complement-dependent cytotoxicity (CDC) [39]. These functions may be irrespective of the neutralization capacity of antibodies as long as they can bind surface antigens on the infected cell. Congruent with this prerequisite, all of the anti-MERS-S antibodies were able to bind cell surface displayed spikes. In addition, our human antibodies were of the IgG1 isotype which was shown to be the most potent human IgG isotype in mice showing efficient binding to all activating mouse Fcγ receptors and induction of ADCC/ADCP with mouse natural killer cells and mouse macrophages [40]. Further research is needed to define the contribution of Fc-functions to antibody-mediated protection from MERS-CoV infection.

We identified human antibodies targeting six distinct epitope classes across different domains of the MERS-CoV spike protein, each of which showing protective activity in mice against lethal MERS-CoV challenge. This discovery holds promise for the development of antibody therapeutics against MERS-CoV infection. Passive immunization with a combination of antibodies targeting different domains and functions of the viral glycoprotein might be more protective against virus infection than single epitope mAb therapy, as was shown for other enveloped RNA viruses [31, 32, 41–43]. Which combination of antibodies provides synergistic protective activity against MERS-CoV and which combination of epitopes is the best to target needs to be further evaluated. In addition, combinations of antibodies targeting distinct epitopes can mitigate viral antigenic escape as has been demonstrated for a number of viruses including MERS-CoV [30, 43–45]. Particularly, the use of (combinations of) antibodies targeting conserved epitopes in the spike glycoprotein that are critical for viral entry may further decrease chances of escape mutants. Such antibodies may as well offer cross-protection against related viruses. Antibodies targeting conserved epitopes in the stem regions of glycoproteins of the enveloped virus including influenza virus and ebolavirus offer protection against a wide range of antigenically distinct variants [46–48]. Likewise, the generation of antibodies targeting the conserved S2 stem of the coronavirus spike protein that can cross-bind spikes of related coronaviruses may even allow the development of mAbs with broad protection against related future emerging coronaviruses. Furthermore, it is important that the antibodies we describe are completely human, which has the important advantage that they can be used several times in the same host without invoking an immune response against the antibody as may be required for people working with camels or infected patients.

Finally, the presence of protective antibody epitopes in multiple spike domains, suggests that multi-domain approaches of spike-based vaccines may provide a broader repertoire of immune responses compared to RBD-focused vaccine antigen and reduce the risk of antigenic escape. This study also defines different correlates of humoral protection, which may need to be considered in the evaluation of vaccine-induced immune responses.

## Materials and methods

***Production of recombinant MERS-CoV S proteins.*** A gene encoding the MERS-CoV spike glycoprotein (EMC isolate; GenBank YP_009047204.1) was synthesized by GenScript USA Inc. and the sequence was codon-optimized to maximize expression in the baculovirus expression system. To produce soluble MERS-S ectodomain, the gene fragment encoding the MERS-CoV S ectodomain (amino acid 19–1262) was cloned in-frame between honeybee melittin (HBM) secretion signal peptide and T4 fibritin (foldon) trimerization domain followed by Strep-tag purification tag in the pFastbac transfer vector (Invitrogen). The furin cleavage site *R*^747^SVR^751^ at the S1/S2 junction was mutated to *K*SVR, to prevent cleavage by furin at this position. The genes encoding MERS-S1 subunit (amino acid 19-748), MERS-S2 ectodomain (amino acid 752-1262), MERS-S1^A^ (amino acid 19-357), and MERS-S1^B^ [358–588] were cloned in-frame between the HBM secretion signal peptide and a triple StrepTag purification tag in the pFastbac transfer vector. Generation of bacmid DNA and recombinant baculovirus was performed according to protocols from the Bac-to-Bac system (Invitrogen) and expression of MERS-CoV S variants was performed by infection of recombinant baculovirus of Sf-9 cells. Recombinant proteins were harvested from cell culture supernatants 3 days post infection and purified using StrepTactin sepharose affinity chromatography (IBA). The soluble MERS-S ectodomain used for immunization was produced in the *Drosophila* expression system as described before [7], by cloning the gene insert from the pFastbac transfer vector into the pMT expression vector (Invitrogen, Thermo Fisher Scientific, the Netherlands). Production of recombinant MERS-S1 (amino acid 1-747) used for immunization, and of soluble DPP4 was described previously [15]. In brief, the MERS-S1 (amino acid 1-747) encoding sequence was C-terminally fused to a gene fragment encoding the Fc region of human IgG and cloned into the pCAGGS mammalian expression vector, expressed by plasmid transfection in HEK-293 T cells, and affinity purified from the culture supernatant using Protein-A affinity chromatography. The Fc part of S1-Fc fusion protein was proteolytically removed by thrombin following Protein-A affinity purification using the thrombin cleavage site present at the S1-Fc junction. The sequence encoding the human DPP4 ectodomain (amino acid 39–766) N-terminally fused to the Streptag purification tag was cloned into the pCAGGS vector, expressed by plasmid transfection of HEK-293 T cells and purified from the cell culture supernatant using StrepTactin sepharose affinity chromatography. Production of lumazine synthase (LS) nanoparticles displaying the MERS-CoV spike domain S1^A^ (S1^A^-LS) has been described previously [16]. In brief, the MERS-S1^A^ encoding sequence (residues 19–357) was N-terminally fused to a CD5 signal peptide sequence, followed by a Streptag purification tag sequence (IBA) and C-terminally fused to the lumazine synthase-encoding sequence from *Aquifex aeolicus* (GenBank WP_010880027.1) via a Gly-Ser linker, and subsequent cloned into the pCAGGS vector, expressed in HEK-293 T cells and purified from the cell culture supernatant using StrepTactin affinity chromatography.

***Production of recombinant monoclonal antibodies.*** For recombinant mAb production, cDNA’s encoding the variable heavy (VH) and light (VL) chain regions of anti-MERS-S H2L2 mAbs were cloned into expression plasmids containing the human IgG1 heavy chain and Ig kappa light chain constant regions, respectively (Invivogen). Both plasmids contain the interleukin-2 signal sequence to enable efficient secretion of recombinant antibodies. Synthetic VH and VL gene fragments of the benchmark antibody (MERS-CTRL) were synthezised based on previously described sequences for the MERS-S monoclonal antibody “H1H15211P” [34]. Recombinant human anti-MERS-S antibodies were produced in HEK-293 T cells following transfection with pairs of the IgG1 heavy and light chain expression plasmids according to protocols from Invivogen. Antibodies were purified from tissue culture supernatants using Protein-A affinity chromatography. Purified antibodies were stored at 4°C until use.

***Generation of anti-MERS-S H2L2 mAbs.*** Two groups of six H2L2 mice were immunized in two weeks intervals six times with purified MERS-S1 (group I) and MERS-S ectodomain followed by MERS-S2 ectodomain (group II) as outlined in Figure 1(A). Antigens were injected at 20 μg/mouse using Stimune Adjuvant (Prionics) freshly prepared according to the manufacturer instruction for the first injection, while boosting was done using Ribi (Sigma) adjuvant. Injections were done subcutaneously into the left and right groin each (50 μl) and 100 μl intraperitoneally. Four days after the last injection, spleen and lymph nodes are harvested, and hybridomas made by a standard method using SP 2/0 myeloma cell line (ATCC#CRL-1581) as a fusion partner. Hybridomas were screened in antigen-specific ELISA and those selected for further development, subcloned and produced on a small scale (100 ml of medium). For this purpose, hybrydomas are cultured in serum- and protein-free medium for hybridoma culturing (PFHM-II (1X) Gibco) with the addition of non-essential amino acids (100× NEAA, Biowhittaker Lonza Cat BE13-114E). Antibodies were purified from the cell supernatant using Protein-G affinity chromatography. Purified antibodies were stored at 4°C until use.

***MERS-S pseudotyped virus neutralization assay*.** Production of VSV pseudotyped with MERS-S was performed as described previously with some adaptations [49]. Briefly, HEK-293 T cells were transfected with a pCAGGS expression vector encoding MERS-S carrying a 16-a.a. cytoplasmic tail truncation. One day post transfection, cells were infected with the VSV-G pseudotyped VSVΔG bearing the firefly (*Photinus pyralis*) luciferase reporter gene [49]. Twenty four hours later, MERS-S-VSVΔG pseudotypes were harvested and titrated on African green monkey kidney Vero cells. In the virus neutralization assay, MERS-S mAbs were serially diluted at two times the desired final concentration in DMEM supplemented with 1% foetal calf serum (Bodinco), 100 U/ml Penicillin and 100 µg/ml Streptomycin. Diluted mAbs were incubated with an equal volume of MERS-S-VSVΔG pseudotypes for 1 h at room temperature, inoculated on confluent Vero monolayers in 96-well plated, and further incubated at 37°C for 24 h. Luciferase activity was measured on a Berthold Centro LB 960 plate luminometer using D-luciferin as a substrate (Promega). The percentage of infectivity was calculated as the ratio of luciferase readout in the presence of mAbs normalized to luciferase readout in the absence of mAb. The half maximal inhibitory concentrations (IC_50_) were determined using 4-parameter logistic regression (GraphPad Prism v7.0).

***MERS-CoV neutralization assay.*** Neutralization of authentic MERS-CoV was performed using a plaque reduction neutralization test (PRNT) as described earlier [50]. In brief, mAbs were two-fold serially diluted and mixed with MERS-CoV for 1 h. The mixture was then added to Huh-7 cells and incubated for 1 hr, after which the cells were washed and further incubated in medium for 8 h. Subsequently, the cells were washed, fixed, permeabilized and the infection was detected using immunofluorescent staining. The PRNT titre was determined as the highest mAb dilution resulting in *a* > 50% reduction in the number of infected cells (PRNT_50_).

***ELISA analysis of MERS-CoV S binding by antibodies.*** NUNC Maxisorp plates (Thermo Scientific) were coated with the indicated MERS-CoV antigen at 100 ng /well at 4°C overnight. Plates were washed three times with Phosphate Saline Buffer (PBS) containing 0.05% Tween-20 and blocked with 3% Bovine Serum Albumin (BSA) in PBS containing 0.1% Tween-20 at room temperature for 2 h. Four-folds serial dilutions of mAbs starting at 10 µg/ml (diluted in blocking buffer) were added and plates were incubated for 1 h at room temperature. Plates were washed three times and incubated with HRP-conjugated goat anti-human secondary antibody (ITK Southern Biotech) diluted 1:2000 in blocking buffer for one hour at room temperature. HRP activity was measured at 450 nm using tetramethylbenzidine substrate (BioFX) and an ELISA plate reader (EL-808, Biotek).

***ELISA analysis of receptor binding inhibition by antibodies.*** Recombinant soluble DPP4 was coated on NUNC Maxisorp plates (Thermo Scientific) at 4°C overnight. Plates were washed three times with PBS containing 0.05% Tween-20 and blocked with 3% BSA in PBS containing 0.1% Tween-20 at room temperature for 2 h. Recombinant MERS-CoV S ectodomain and serially diluted anti-MERS mAbs were mixed for 1 h at RT, added to the plate for 1 h at room temperature, after which plates were washed three times. Binding of MERS-CoV S ectodomain to DPP4 was detected using HRP-conjugated anti-StrepMAb (IBA) that recognizes the Streptag affinity tag on the MERS-CoV S ectodomain. Detection of HRP activity was performed as described above.

***Antibody competition assay.*** Competition among mAbs for binding to the same epitope on MERS-CoV S was determined using Bio-Layer Interferometry (BLI) on Octet QK (Pall ForteBio) at 25°C. All reagents were diluted in PBS. The assay was performed following these steps: (1). anti-Strep mAb (50 μg/ml) was coated on Protein A biosensor (Pall ForteBio) for 30 mins, (2) blocking of sensor with rabbit IgG (50 μg/ml) for 30 mins, (3). Recombinant Strep-tagged MERS-CoV S ectodomain (50 μg/ml) was immobilized to the sensor for 15 mins (4). Addition of mAb #1 (50 μg/ml) for 15 min to allow saturation of binding to the immobilized antigen, (5) Addition of a mAb #2 (50 μg/ml) for 15 mins. The first antibody (mAb #1) was taken along to verify the saturation of binding. A 5-minutes washing step in PBS was included in between steps.

***Binding kinetics and affinity measurements.*** Binding kinetics and affinity of mAbs to the MERS-S ectodomain was measured by BLI using the Octet QK at 25°C. The optimal loading concentration of anti-MERS-S mAbs onto anti-human Fc biosensors (Pall ForteBio) was predetermined to avoid saturation of the sensor. The kinetic binding assay was performed by loading anti-MERS mAb at optimal concentration (42 nM) on anti-human Fc biosensor for 10 mins. Antigen association step was performed by incubating the sensor with a range of concentrations of the recombinant MERS-S ectodomain (200–67 to 22–7.4 nM) for 10 min, followed by a dissociation step in PBS for 60 min. The kinetics constants were calculated using 1:1 Langmuir binding model on Fortebio Data Analysis 7.0 software.

***Hemagglutination inhibition assay.*** The potency of mAbs to inhibit hemagglutination by MERS-S1^A^-displaying lumazine synthase nanoparticles (S1^A^-LS) was performed as described previously [16], with slight modification. Two-folds serial dilutions of S1^A^-LS in PBS containing 0.1% bovine serum albumin (BSA) were mixed with 0.5% human erythrocyte in V-bottom 96-well plate (Greiner Bio-One), and incubated at 4°C for 2 h. The hemagglutination titre was scored, and the concentration of S1^A^-LS that resulted in 8 hemagglutination units was determined. Subsequently, two-fold serial dilutions of anti-MERS-S mAbs in PBS containing 0.1% BSA were mixed with S1^A^-LS (8 hemagglutination units) in a V-bottom 96-well plate. After 30 mins incubation at room temperature, human erythrocytes were added to a final concentration of 0.5% (v/v). The mixture was incubated at 4°C for 2 h and the hemagglutination inhibition activity by anti-MERS-S mAbs was scored.

***Fusion inhibition assay.*** Huh-7 cells were seeded with a density of 10^5^ cells per ml. After reaching 70–80% confluency, cells were transfected with expression plasmid encoding full length MERS-CoV S fused to Green Fluorescence Protein (GFP) using jetPRIME^®^ (Polyplus transfection, New York, USA; cat no. 114-07). The furin recognition site in the MERS-CoV S was mutated to inhibit the cleavage of protein. Two days post transfection, cells were treated with 10 μg/ml trypsin (to activate MERS-CoV spike fusion function) in the presence or absence of 10 μg/ml anti-MERS-S mAbs. After incubation at 37°C for 2 h, the cells were fixed by incubation with 4% paraformaldehyde in PBS for 20 min at room temperature and stained for nuclei with 4,6-diamidino-2-phenylindole (DAPI). Cells expressing MERS-CoV S were detected by fluorescence microscopy using the C-terminally appended GFP and MERS-CoV S-mediated cell–cell fusion was observed by the formation of (fluorescent) multi-nucleated syncytia. The fluorescence images were recorded using the EVOS FL fluorescence microscope (Thermo Fisher Scientific, the Netherlands).

***Antibody-mediated protection of mice challenged with MERS-CoV.*** In vivo efficacy of mAbs specific for the S protein of MERS-CoV, and of an isotype matched negative control mAbs was evaluated in the protection of the transgenic mouse model K18 TghDpp4 expressing the receptor for the human MERS-CoV [36] susceptible to the virulent virus. MERS-CoV intranasal infection of these transgenic mice expressing human DPP4 causes a lethal disease associated with encephalitis, lung mononuclear cell infiltration, alveolar oedema, and microvascular thrombosis, with airways generally unaffected [36]. To test the prophylactic efficacy of mAbs in vivo groups of 5 mice, 20–30 weeks old, were given 1.8 mg of the antibody per kg mouse by intraperitoneal injection, 6 h before intranasal infection with a lethal dose of MERS-CoV (EMC isolate; 5 × 10^3^ pfu/mouse, challenge dose resulting in consistently lethal infection in untreated mice). Whereas the administered dose was consistently lethal for all mice that received the isotype control antibody, mice that received the best virus neutralizing antibodies were fully protected.

## Supplementary Material

Supplemental Material
