# Supplementary material for: Towards a solution to MERS: protective human monoclonal antibodies targeting different domains and functions of the MERS-coronavirus spike glycoprotein
Source: Emerg Microbes Infect. 2019 Apr 2;8(1):516–30. doi: 10.1080/22221751.2019.1597644 (PMC6455120; doi:10.1080/22221751.2019.1597644)
Supplement: Supplemental Material [file TEMI_A_1597644_SM3122.zip › Supplementary Material/Legends Supplementary Figures.docx]

**Legends Supplementary Figures**

**Figure S1. SDS-PAGE analysis of purified, recombinant MERS-CoV S antigens.** SDS-PAGE analysis of purified MERS-CoV S antigens (2 μg each) used for immunization of H2L2 transgenic mice **(A)** or for ELISA-based screening of antibodies **(B)**.

**Figure S2. Characterization of anti-MERS-S H2L2 antibodies. (A)** Characterization of anti-MERS-S1 H2L2 antibodies from antibody-containing hybridoma supernatants. First, antibody-containing hybridoma supernatants (selected based on an initial MERS-S1 ELISA screen) were screened for ELISA-reactivity to S1 or individual domains of S1 (S1^A^, S1^B^ or S1^CD^) (upper four panels). Second, virus neutralization by S1-reactive hybridoma supernatants was analysed using the luciferase-encoding MERS-S pseudotyped VSV particles (bottom panel). **(B)** Binding competition of anti-MERS-S neutralizing H2L2 mAbs using bio-layer interferometry. Immobilized MERS-S^ecto^ antigen was saturated in binding with a given anti-MERS-S H2L2 mAb (step 1) and then exposed to binding by a second H2L2 mAb (step 2). Additional binding of the second antibody indicates the presence of an unoccupied epitope, whereas lack of binding indicates epitope blocking by the first antibody. As a control, the first mAb was also included in the second step to check for self-competition. **(C)** Neutralizing activity of purified H2L2 antibodies was assessed using MERS-S pseudotyped VSV and half-maximal inhibitory concentrations (IC_50_; μg/ml) are shown. H2L2 antibodies were purified from hybridoma supernatants with neutralizing activity that were reactive to MERS-S1^B^. In addition, H2L2 antibodies were purified from hybridoma supernatants showing MERS-S2 and MERS-S1^A^ domain reactivity. Selection of lead mAbs was based on their potency to neutralize MERS-CoV relative to other mAbs within an epitope group (epitope groups are marked by shaded blocks), and on their unique VH and VL region sequences. Eight monoclonal antibodies (shown by red arrows) with epitopes distributed throughout different domains of the MERS-CoV spike protein were selected as lead antibodies for further detailed biophysical and functional characterization.

**Figure S3. SDS-PAGE analysis of purified monoclonal antibodies.** Two microgram of the eight lead anti-MERS-S mAbs, an anti-MERS control antibody and an isotype control antibody were analysed by SDS-PAGE. All antibodies have the human IgG1 isotype background, were expressed in human HEK-293T cells and purified using Protein-A affinity purification.

**Figure S4. MERS-S-specific mAbs bind MERS-S^ecto^ with high affinity.** The binding kinetics and affinity of anti-MERS-S mAbs and DPP4 receptor to recombinant soluble MERS-S^ecto^ was measured by Bio-layer interferometry. Antibodies and receptor were immobilized on the sensor surface, followed by injection of the MERS-S^ecto^ at 200, 67, 22 and 7.4 nM concentrations (represented by the blue, red, green purple lines, respectively). The kinetics constants were calculated using 1:1 Langmuir binding model on Fortebio Data Analysis 7.0 software. The binding rate constant *k_on_* (M^−1^ sec^−1^), the dissociation constant *k_off_* (sec^−1^) and the equilibrium dissociation constant *K_D_* (M; *K_D_* = *k_off_/k_on_*) are shown below the sensograms.

**Figure S5. Binding of anti-MERS-S mAbs to cells expressing MERS-CoV spike protein detected by flow cytometry.** Overlay histograms showing the binding of anti-MERS-S mAbs to MERS-CoV S expressing Huh-7 cells by flow cytometric analysis. Huh-7 cells were seeded with density of 2.5×10^5^ cells per ml in a T75 flask. After reaching 70~80% confluency, cells were transfected with expression plasmid encoding full length MERS-S - C-terminally fused to the green fluorescence protein (GFP) - using jetPRIME® (Polyplus transfection). The furin recognition site in the MERS-S was mutated (R^751^S) to inhibit the cleavage of protein. Two days post transfection, cells were dissociated by cell dissociation solution (Sigma-aldrich, Merck KGaA; cat. no. C5914). Single cell suspensions in FACS buffer were centrifuged at 400×g for 10 min. Cells were then fixed by incubation with 4% paraformaldehyde in PBS for 15 min at room temperature. After centrifugation at 600×g for 10 min, paraformaldehyde was removed. Follwed by a washing step in Phosphate Saline Buffer (PBS), cells were blocked using 10% Normal Goat Serum (Gibco, Thermo Fisher Scientific, the Netherlands) diluted in PBS for 45 min at room temperature. Surface staining of MERS-S was performed by incubation of cells with anti MERS-CoV mAbs at concentration of 5 µg/ml for 1 h at room temperature, followed by incubation with Alexa Fluor 649 conjugated goat anti-human IgG antibodies (Invitrogen, Thermo Fisher Scientific) for 45 min at room temperature. Cells were subjected to flow cytometric analysis with a FACS-Calibur flow cytometer (BD Biosciences) and gated for GFP expression. The results were analysed by FlowJo (version 10), and mean fluorescence intensities (MFIs) were calculated.

**Figure S6. Binding of anti-MERS-S mAbs to cell surface expressed MERS-CoV spike protein detected by immunofluorescence microscopy.** Huh-7 cells were seeded with density of 10^5^ cells per ml. After reaching 70~80% confluency, cells were transfected with expression plasmid encoding full length MERS-S - C-terminally fused to the green fluorescence protein (GFP) - using jetPRIME® (Polyplus transfection, New York, USA). The furin recognition site in the MERS-S was mutated (R^751^S) to inhibit the cleavage of protein. Two days post transfection, cells were fixed by incubation with 4% paraformaldehyde in PBS for 20 min at room temperature. After extensive washing in PBS containing 0.05% Tween-20, cells were blocked using 10% Fetal Calf Serum (Bodinco B.V) diluted in PBS with 0.05% Tween-20 for 20 min at room temperature. MERS-S cell surface staining was performed by incubation of MERS-S transfected cells with anti-MERS-S mAbs at concentration of 1.25 µg/ml for 1 h at room temperature, followed by incubation with AlexaFluor 568 conjugated goat anti-human IgG (H+L) secondary antibody (Invitrogen, Thermo Fisher Scientific, the Netherlands) for 1 h at room temperature. Isotype control was included to check for background signal. Antibody binding to cells was detected by fluorescence microscopy using Alexa Fluor 568 conjugated goat anti-human IgG antibodies (red channel). MERS-S-GFP transfected cells were detected by GFP fluorescence (green channel). Staining of nuclei was performed using 4,6-diamidino-2-phenylindole (DAPI) (blue channel). Fluorescence images were recorded using EVOS FL fluorescence microscope (Thermo Fisher Scientific). Panels on the right represent overlay profiles of the red, green and blue channels.
