# Supplementary figures and images for: Towards a solution to MERS: protective human monoclonal antibodies targeting different domains and functions of the MERS-coronavirus spike glycoprotein
Source: Emerg Microbes Infect. 2019 Apr 2;8(1):516–30. doi: 10.1080/22221751.2019.1597644 (PMC6455120; doi:10.1080/22221751.2019.1597644)

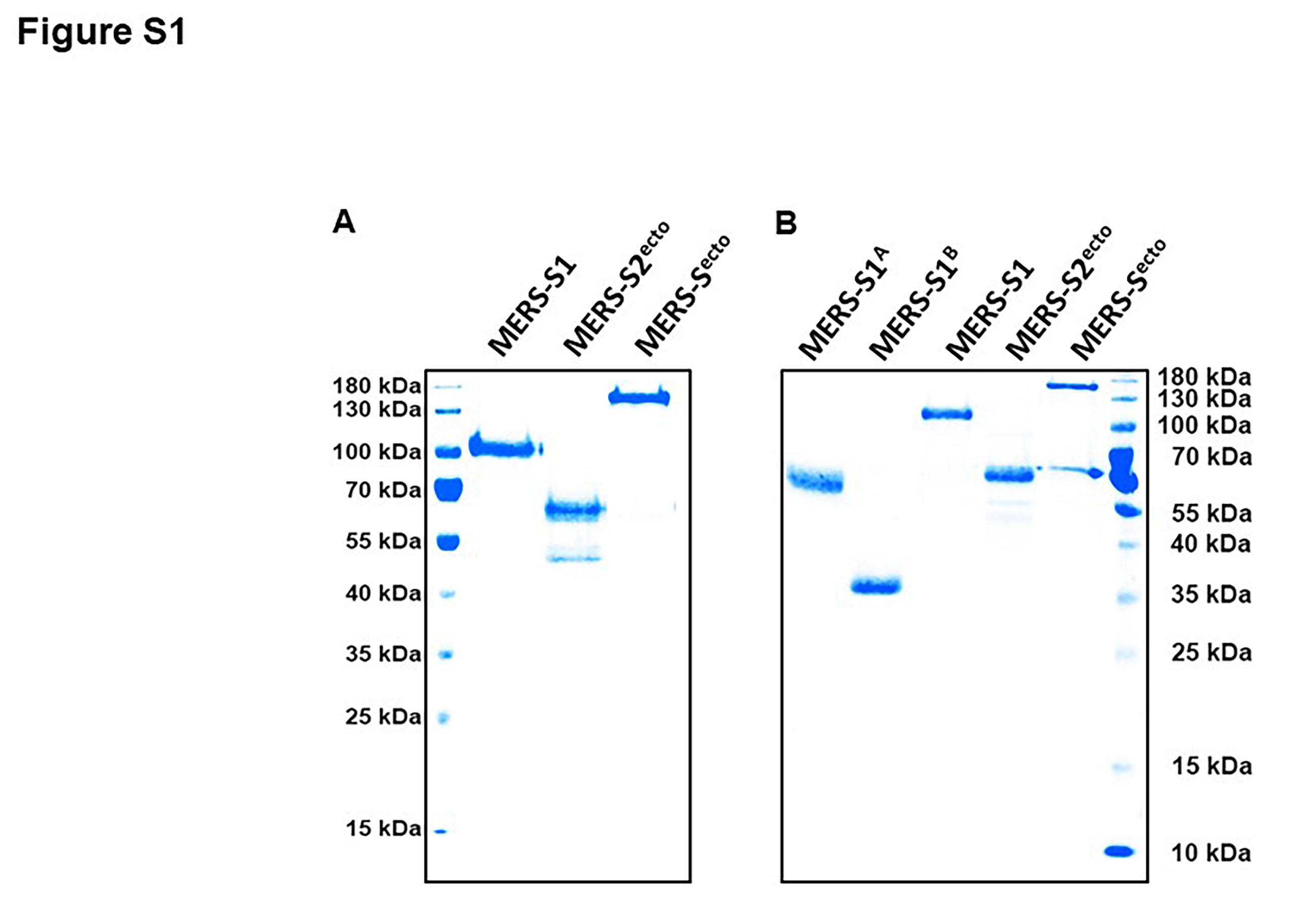

Supplement: Supplemental Material [file TEMI_A_1597644_SM3122.zip › Supplementary Material/Figure S1.jpg]

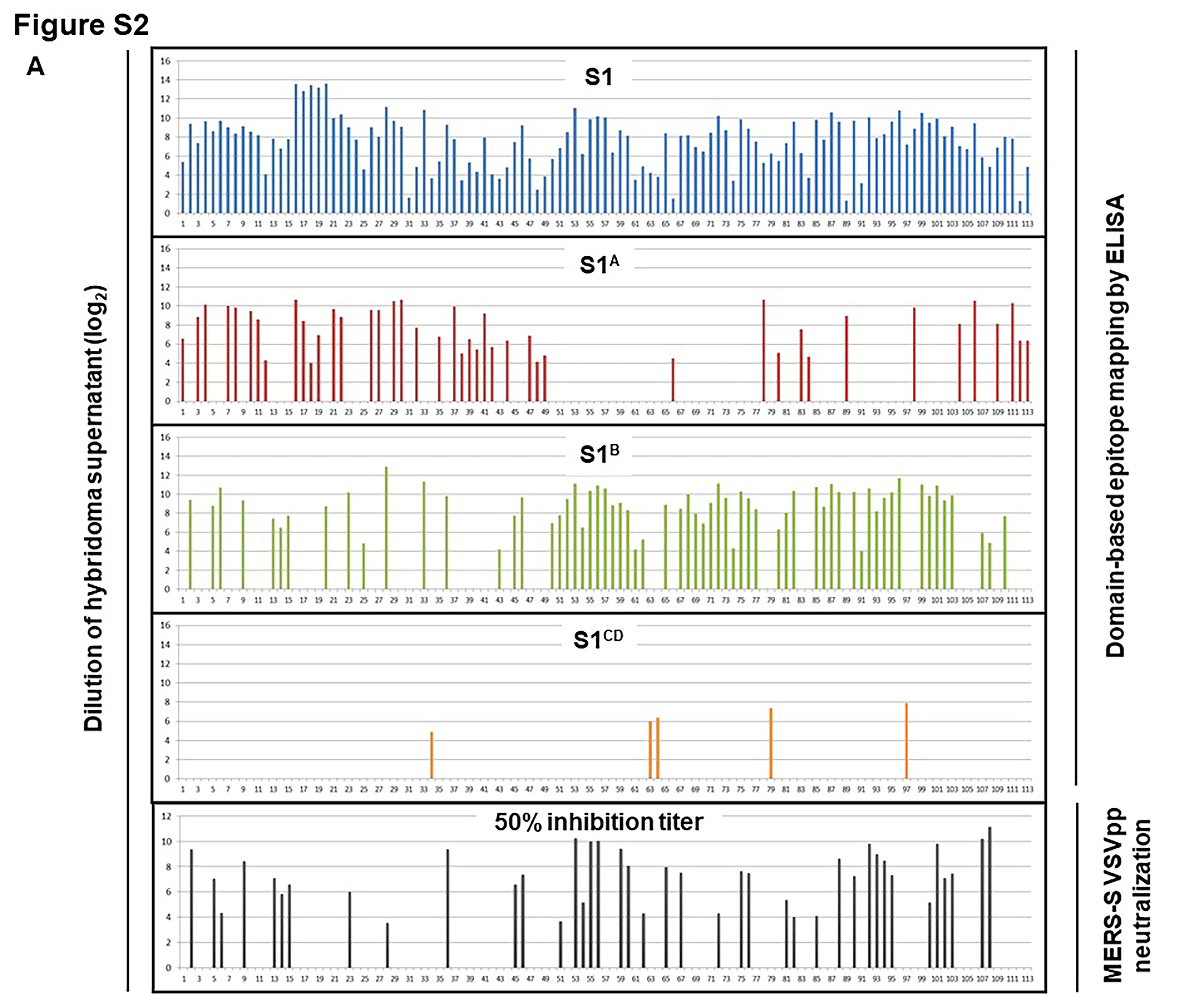

Supplement: Supplemental Material [file TEMI_A_1597644_SM3122.zip › Supplementary Material/Figure S2A.jpg]

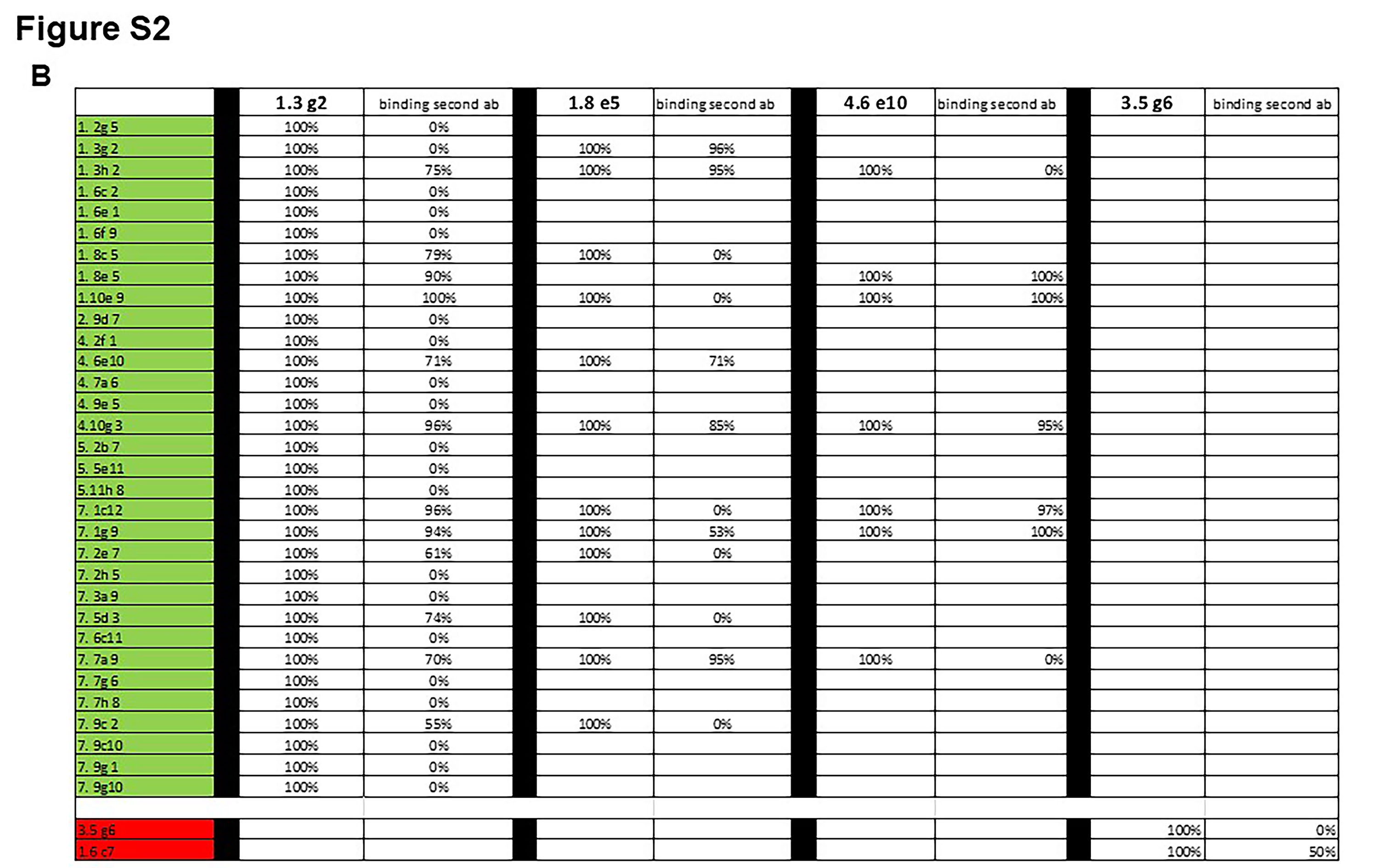

Supplement: Supplemental Material [file TEMI_A_1597644_SM3122.zip › Supplementary Material/Figure S2B.jpg]

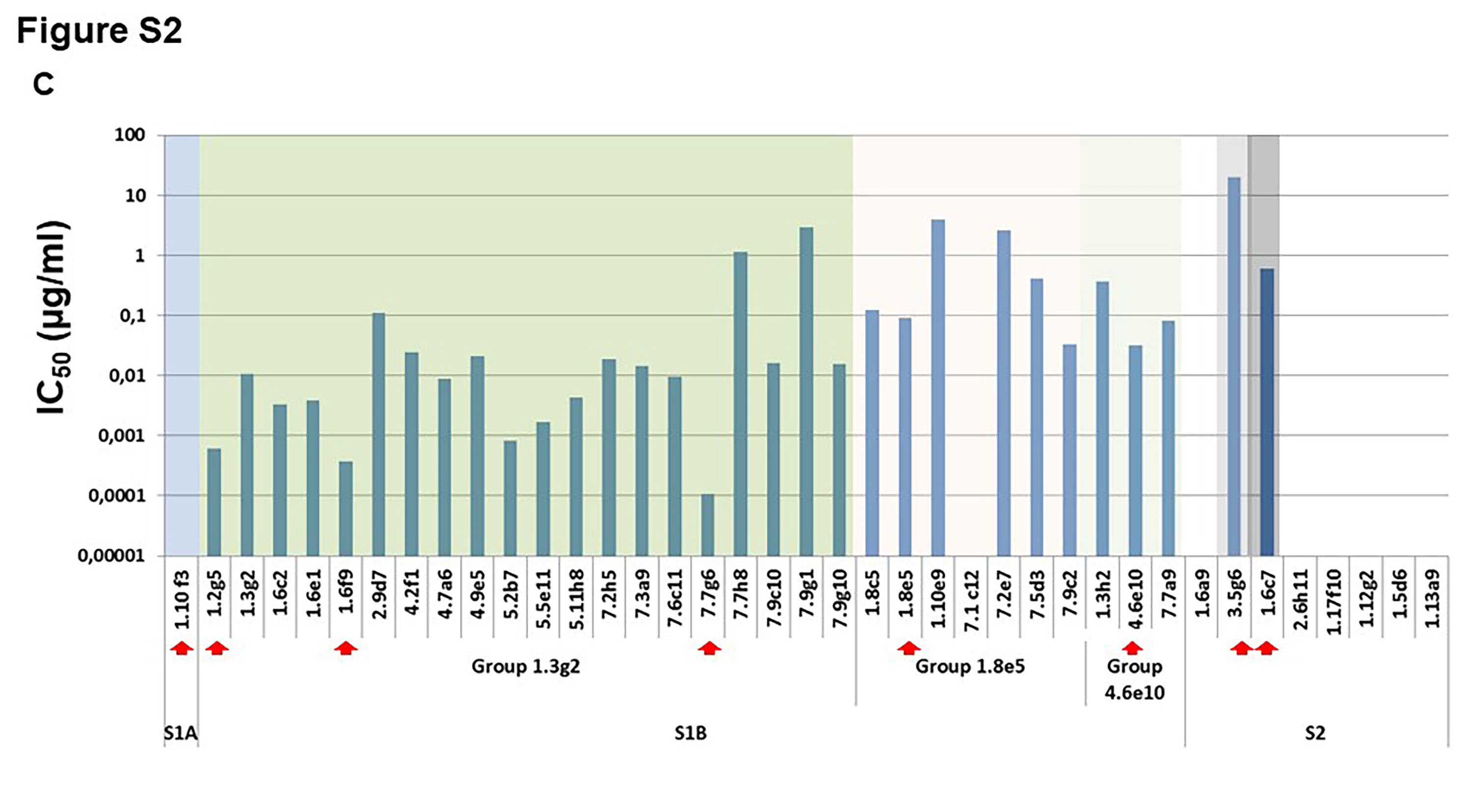

Supplement: Supplemental Material [file TEMI_A_1597644_SM3122.zip › Supplementary Material/Figure S2C.jpg]

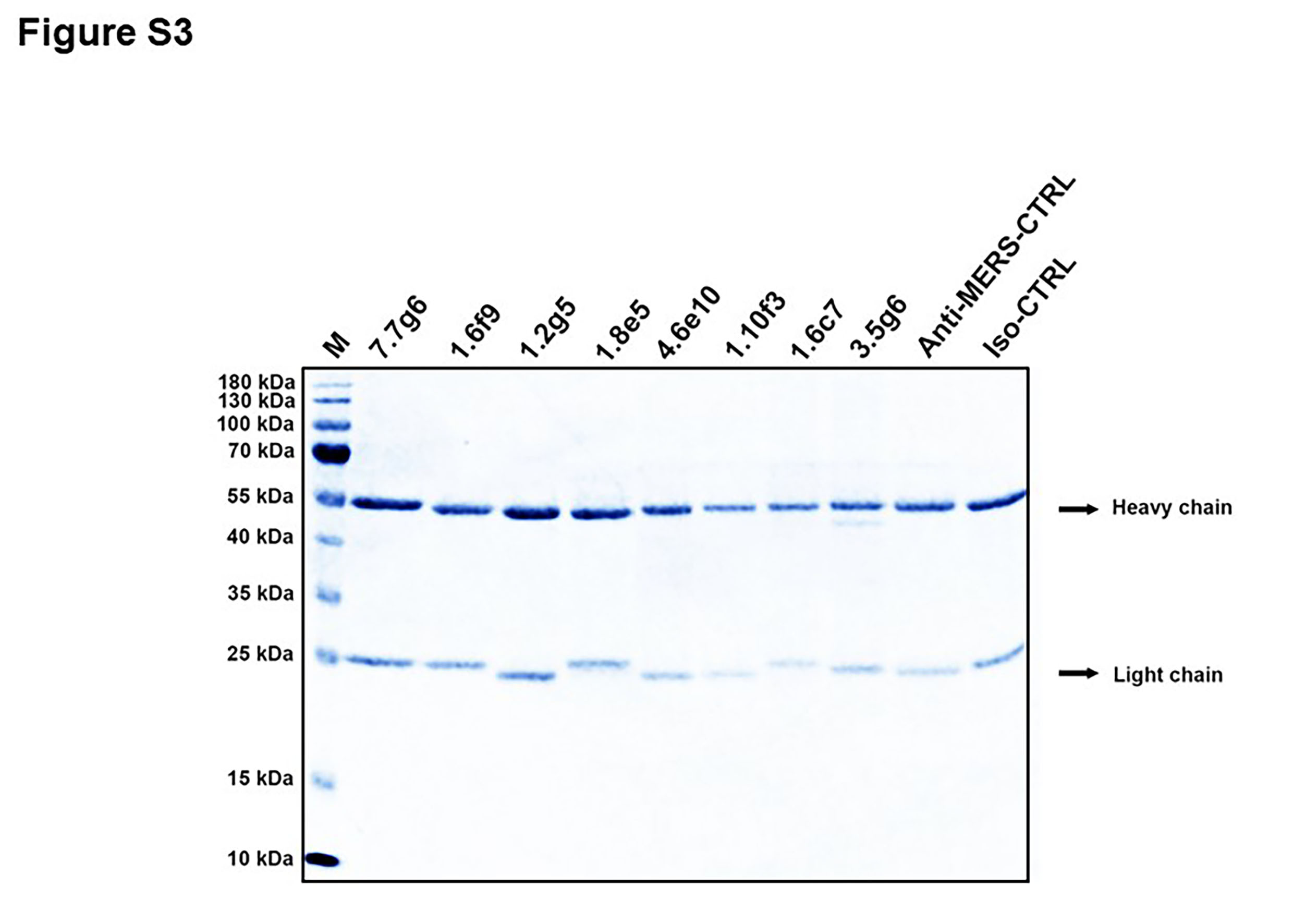

Supplement: Supplemental Material [file TEMI_A_1597644_SM3122.zip › Supplementary Material/Figure S3.jpg]

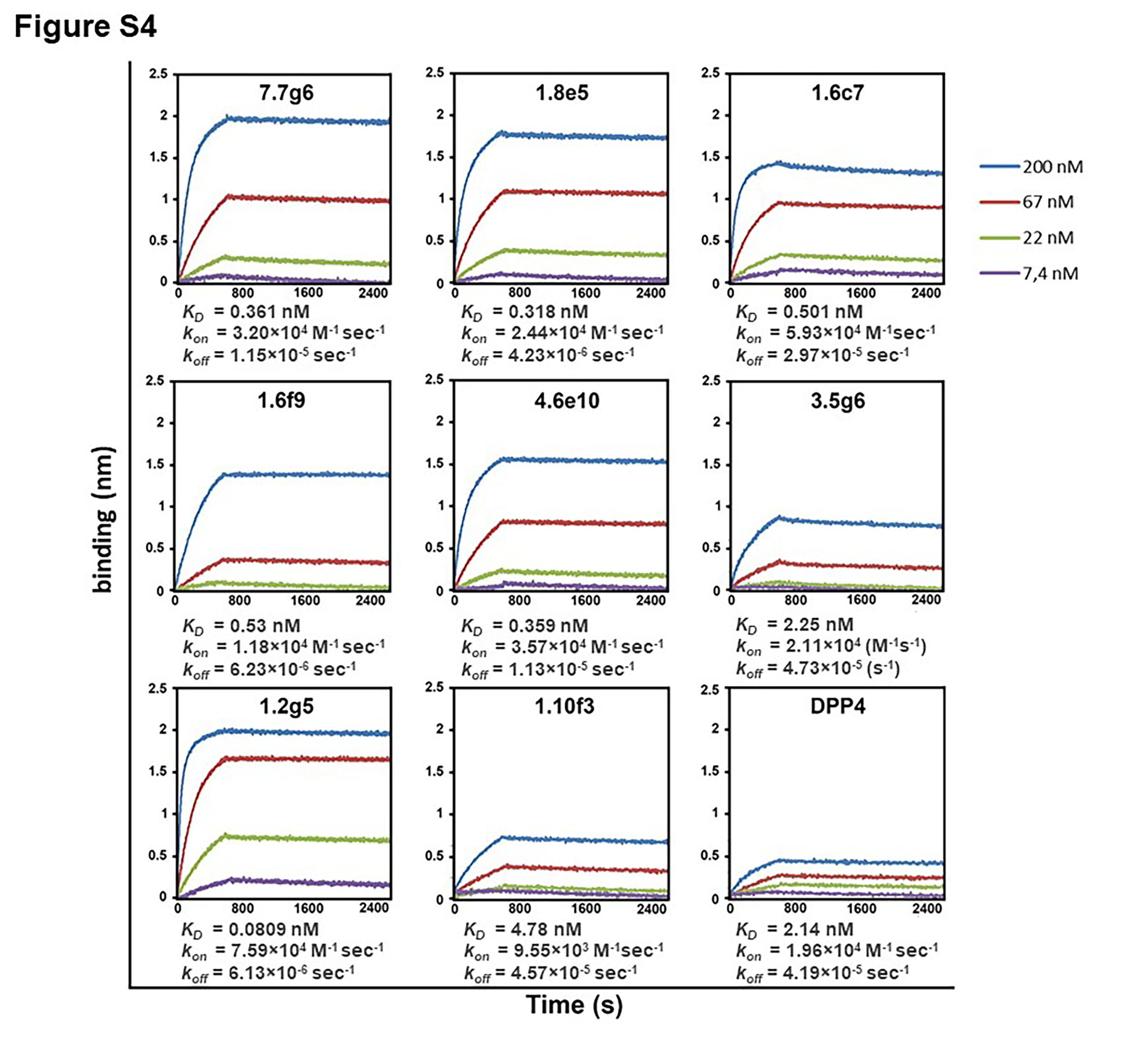

Supplement: Supplemental Material [file TEMI_A_1597644_SM3122.zip › Supplementary Material/Figure S4.jpg]

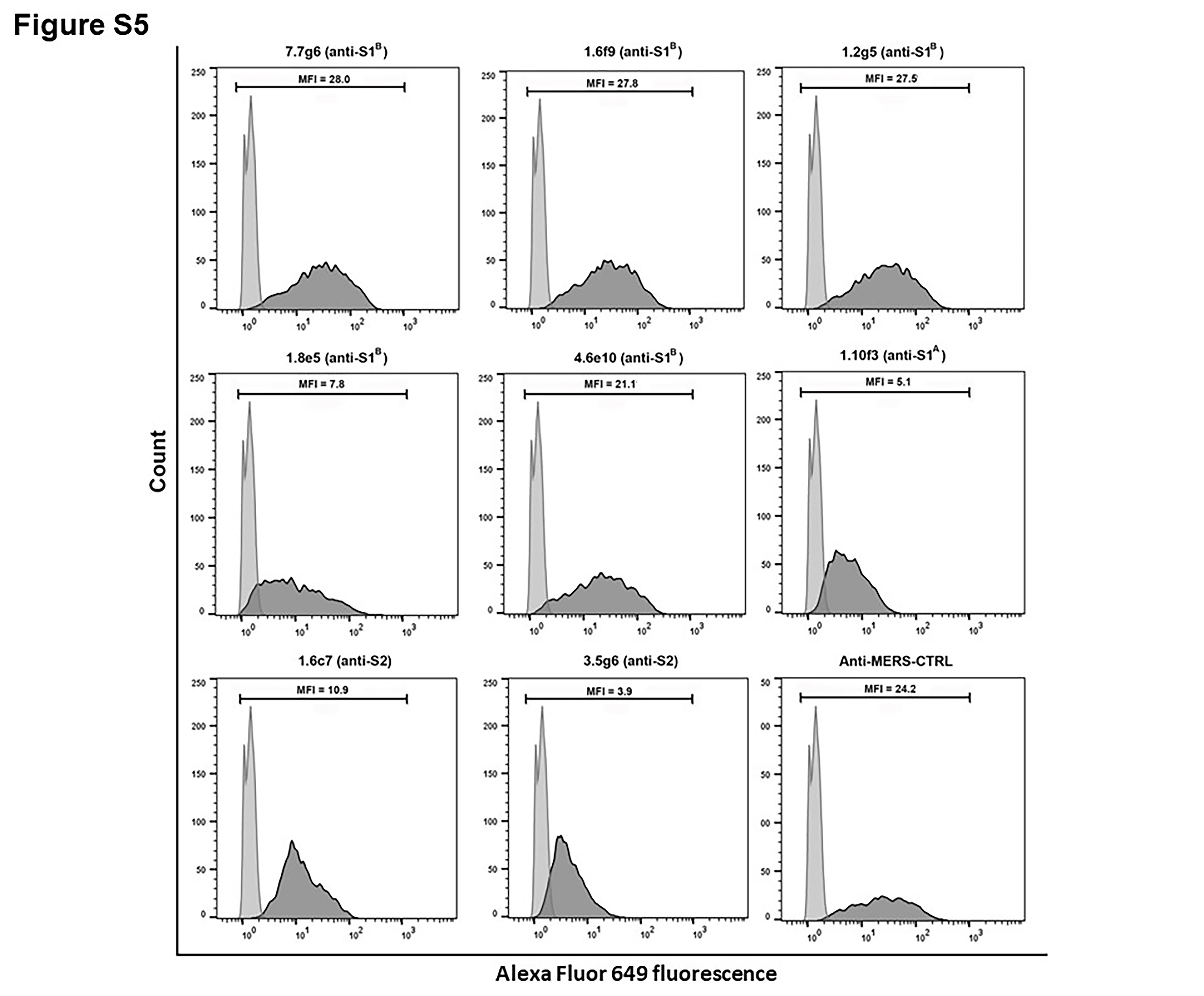

Supplement: Supplemental Material [file TEMI_A_1597644_SM3122.zip › Supplementary Material/Figure S5.jpg]
